# Autotransplantation of parathyroid grafts into the tibialis anterior muscle after parathyroidectomy: a novel autotransplantation site

**DOI:** 10.1186/s12893-015-0098-x

**Published:** 2015-10-15

**Authors:** Chrysanthi Anamaterou, Matthias Lang, Simon Schimmack, Gottfried Rudofsky, Markus W. Büchler, Hubertus Schmitz-Winnenthal

**Affiliations:** Department of Medicine I and Clinical Chemistry, University of Heidelberg, Im Neuenheimer Feld 410, 69120 Heidelberg, Germany; Department of General, Visceral and Transplantation Surgery, University of Heidelberg, Im Neuenheimer Feld 110, 69120 Heidelberg, Germany; Department of Internal Medicine, Kantonsspital Olten, Baslerstrasse 150, 4600 Olten, Switzerland

**Keywords:** Renal hyperparathyroidism, Parathyroidectomy, Autotransplantation, Casanova-test

## Abstract

**Background:**

Surgical management of renal secondary hyperparathyroidism (sHPT) is varying. Total parathyroidectomy with heterotopic autotransplantation (TPTX + AT) is one of the standard surgical procedures in sHPT, but there is no consensus about the optimal site for graft insertion. At the surgical department of the University Hospital of Heidelberg we prefer the autotransplantation into the tibialis anterior muscle. The aim of this study was to assess the long-term function of the auto-transplanted parathyroid tissue in this type of surgical procedure.

**Methods:**

The function of the autograft of 42 patients was assessed 8.2 ± 2.5 years after surgery, using a modified Casanova-test of the leg bearing the parathyroid tissue. Ischemic blockage was induced by tourniquet and the levels of parathyroid hormone (PTH) were assessed during the test.

**Results:**

At the point of assessment, the ischemic blockage led to a significant reduction in the concentration of PTH (≥50 % of the baseline value) in 19 patients (45 %) indicating well-functioning autografts. In 11 patients (26 %), ischemic blockage did not cause any change in the concentration of PTH (≤20 % of the baseline value), indicating functioning residual parathyroid tissue from another site. The source of PTH production was classified as unidentifiable in five patients (12 %). Two patients had developed graft-dependent recurrent HPT (5 %) without therapeutic consequences and three patients suffered from persistent symptomatic hypoparathyroidism (7 %).

**Conclusions:**

These results indicate that TPTX + AT into the tibialis anterior muscle is a successful surgical treatment for renal HPT and that the modified Casanova-test is a suitable diagnostic tool for autografts function.

## Background

Secondary hyperparathyroidism (sHPT) is a frequent complication of chronic renal insufficiency (CRI), especially end-stage renal disease (ESRD) [1, 2]. Advances in the medical treatment of sHPT have reduced the need for surgery, however 5–10 % of patients still require parathyroidectomy [2–4]. Surgical management of sHPT is varying [2, 3]. It can be performed using either subtotal parathyroidectomy or total parathyroidectomy (TPTX) with or without autotransplantation (AT) [2, 3, 5, 6]. AT of parathyroid grafts into the sternocleidomastoid muscle [7, 8] or the subcutaneous abdominal adipose tissue have been widely used [9], however Wells et al. have demonstrated that the forearm muscle can be more advantageous [10–12]. Subcutaneous pre-sternal transplantation [13], subcutaneous injection or transplantation in the forearm [14, 15] and intramuscular injection in the deltoid [16], have also been described.

At the University Hospital of Heidelberg, the preferred method is TPTX with AT of half of the parathyroid gland into the (right) tibialis anterior muscle. This is to preserve the forearm if any additional shunt replacement and/or revision in patients with sHPT become necessary. This method also allows further surgeries under local anesthesia when recurrent graft-dependent HPT appears [2, 6]. Furthermore the function of the remnant can be observed by a Casanova-test; which is not the case for in patients with remnants in the neck or abdominal wall [17, 18]. The effectiveness of this method, particularly with respect to the location of AT, has yet to be described.

The Casanova-test was developed to establish graft dependent recurrence [17, 19]. A simplified version of the test, without intravenous regional analgesia (SCAT), has been shown to distinguish the site of recurrence in 90 % of patients, when comparing to the measurement of parathyroid hormone (PTH) levels at 10 min of suprasystolic exclusion of the grafted arm to preischemic values [18].

Currently, there is no accepted standard for either the optimal site of AT or for defining a fully functional autograft. The objective of this study is to evaluate the feasibility of the new AT site and determine the function of the parathyroid autografts by means of the modified Casanova-test.

## Methods

### Study design and study population

Between 2002 and 2010, AT of parathyroid tissue into the tibialis anterior muscle was the standard approach applied in renal patients who were undergoing parathyroidectomy at the University Hospital of General, Visceral and Transplantation Surgery of Heidelberg, Germany. Forty-two patients after TPTX with AT into the tibialis anterior muscle were retrospectively examined on average 8.2 ± 2.5 years after surgery. Patients with primary hyperparathyroidism or recurrent/ persistent disease and patients under 18 years of age were not considered for enrollment. The study population consisted of 26 men (mean age 50.5 ± 12.4 years) and 16 women (mean age 56.1 ± 11.6 years). Until surgery all patients received maximum conservative therapy (vitamin D and/or analogues and phosphate binders) and only five patients received calcimimetic agents (cinacalcet). At presentation one third of the patients were still on dialysis. Written informed consent for participation in the study and publication of acquired data was obtained from all study patients. Ethics approval was provided by the ethics committee of the medical faculty of the University of Heidelberg (Ethics Approval Number S-169/2011).

### Surgical procedure

After removal of all identified parathyroid glands, half of the smallest (0,2–0,5 cm) and most physiological -not hyperplastic- appearing gland was carefully comminuted into 1 mm-thick pieces. After incision in the right lower leg (2 cm), an avascular pocket was created in the tibialis anterior muscle and the pieces of the parathyroid gland were implanted in all 42 patients, marked with a clip, and the muscle pocket was closed with a suture. Additional cervical thymectomy was performed in 13 patients (total or partial); total or subtotal thyroidectomy in five patients and both in one patient. Final histology reports revealed the presence of four parathyroid glands in 36 patients (86 %), three glands in five patients and two glands in one patient. The most common complication was mild postoperative hypocalcemia (mean 1.90 ± 0.15 mmol/l) in 22 patients (52 %). Other complications observed included hungry-bone syndrome (three patients, 7 %), hyperthyroidism (two patients, 5 %), and transient partial recurrent laryngeal nerve palsy (two patients, 5 %).

### Casanova-test

Modified Casanova-test was performed as previously described [17–19]. A Visomat comfort Typ 2040 blood pressure cuff (UEBE Medical GmbH, Wertheim, Germany) was used to measure systemic blood pressure at the left upper arm. After padding the right upper leg with cotton wool, a pneumatic tourniquet (Welch Allyn large adult Nr.12 durable blood pressure cuff or ERKA perfect aneroid extra large cuff) was placed. The tourniquet was inflated between 30 and 50 mmHg over the systolic blood pressure. Lack of pulse of the lower limb was assessed. Ischemia of the lower limb was maintained for 15 min. Blood samples were obtained from the arm before the ischemic period, during the ischemic period (at 5; 10; 15 min) and 10 min after deflating the tourniquet. Intact parathyroid hormone was assessed using the sandwich ELISA (Advia Centaur XP; SIEMENS, Germany). The source of PTH secretion was defined according to the time course of the PTH-levels during ischemia, as previously suggested by Casanova et al. [17]. In brief, a PTH decrease of more than 50 % of the pre-ischemic value (test positive) indicates the source at the tibia, whereas a PTH decrease of less than 20 % of the pre-ischemic value (test negative), indicates the neck or mediastinum. A PTH decrease more than 20 % and less than 50 %, indicates an undefined source of PTH secretion.

All patients underwent additional ultrasonography of the neck and the lower limb, electrocardiography and venous blood sampling (calcium, phosphate, alkaline phosphatase, albumin, creatinine, urea, thyroid hormones). A 99mTc-sestamibi scintigraphy was not performed as all patients were asymptomatic and showed low PTH levels.

### Clinical definitions

Hypocalcemia was defined as a serum calcium level less than 2.1 mmol/l (normal range: 2.1–2.65 mmol/l), severe hypocalcemia was defined as serum calcium less than 1.75 mmol/l with associated symptoms. Severe hypercalcemia was defined as serum calcium levels more than 3.0 mmol/l. Elevated PTH was defined as PTH levels higher than 7.6 pmol/l (normal range: 1.3–7.6 pmol/l) without therapeutic consequences. Recurrent HPT was defined as PTH levels higher than the Kidney Disease Outcomes Quality Initiative (KDOQI) recommended levels ca. 6 months after surgery [20]. Biochemical hypoparathyroidism was defined as insignificant/undetected PTH levels (PTH ≤ 0.3 pmol/l).

### Statistics

Statistical analysis was performed using the *t*-test of unpaired data (Mann–Whitney *t*-test) and/or the Chi-square test with GraphPad Prism 5 (*GraphPad Software Inc., La Jolla, USA*). Significance was defined as *p* < 0.05.

## Results

At the time of examination (in average 8.2 years after surgery), 20 patients had normal PTH levels (48 %, mean 3.6 ± 1.9 pmol/l), eight patients had a mild hypoparathormonemia (19 %, mean 0.8 ± 0.4 pmol/l) and seven patients had a biochemical hypoparathyroidism (17 %). Among those patients with biochemical hypoparathyroidism, three had typical symptoms of hypocalcemia (7 %), but were normocalcemic under daily substitution of calcium and vitamin D. Three patients had a recurrent HPT (7 %) according to the KDOQI-recommended PTH levels [20]. This included two patients after kidney transplantation and one patient on hemodialysis; all three patients were normocalcemic and had no typical signs and symptoms of HPT (generalized bone and muscle pain, numbness, spontaneous fractures, fatigue, mental status changes, pruritus, nephrolithiasis). The former had double the upper KDOQI-recommended PTH levels (66.1 pmol/l) and was the only patient who demonstrated a sonographic enlarged and palpable parathyroid transplant in the tibialis anterior muscle. This patient refused surgical reexploration. The need for therapeutic intervention was stated after the KDOQI criteria or symptoms causing discomfort for the patient. The dose of vitamin D was adjusted and the patients were referred to follow up in the nephrology department. To date there has been no need for revision.

PTH levels showed a significant decrease after surgery (preoperative 84.0 ± 56.2 pmol/l vs. postoperative 1.4 ± 2.4 pmol/l, *p* < 0.0001).

Of the 42 patients, 25 were normocalcemic (60 %, mean 2.34 ± 0.14 mmol/l) and 15 were slightly hypocalcemic (36 %, mean 1.88 ± 0.10 mmol/l). One patient had a mild hypercalcemia (2 %, 2.71 mmol/l) and one a severe hypocalcemia (2 %, 1.63 mmol/l) with normal PTH levels and without typical symptoms.

Calcium levels also showed a significant decrease after surgery (preoperative 2.60 ± 0.24 mmol/l vs. postoperative 2.11 ± 0.34 mmol/l, *p* < 0.0001) (Tables 1 and 2).

**Table 1:** Clinical and biochemical data of the study population

No	Calcium prior to surgery (mmol/l)	PTH prior to surgery (pmol/l)	Calcium at presentation (mmol/l)	PTH at presentation (pmol/l)	Creatinine at presentation (mg/dl)	Casanova-Test prediction	PTX (number of glands)	Transcervical thymectomy	Time after PTX (months)	Dialysis
1	2.40	147	1.89	0.9	1.4	N	4	half	132	No
2	2.52	78.2	1.96	25.2	10.5	U	4	half	48	Yes
3	2.48	27.2	2.33	66.1	6.1	P	3	No	120	Yes
4	2.81	143.9	1.93	4.9	6.5	U	4	No	96	Yes
5	2.57	60.6	2.47	1.8	12.2	P	4	No	48	Yes
6	2,37	17.5	1.90	3.3	8.5	P	4	Yes	84	Yes
7	2.92	149.8	2.62	<0.1	6.1	M	4	Yes	72	Yes
8	2.70	22.4	1.90	2.3	0.9	P	4	No	120	No
9	2.69	15.5	2.29	3.9	1.3	P	4	No	132	No
10	2.73	41.8	1.89	1.6	1.4	U	4	Yes	84	No
11	2.74	56	2.15	10.4	9.1	N	3	half	72	Yes
12	2.44	63.4^a^	2.21	2.7	1.2	N	4	No	96	No
13	2.84	78.1	2.36	2	6.9	P	4	No	84	Yes
14	2.60	55.6	2.40	1.4	1.7	P	5	No	132	No
15	2,59	34.7	2.46	8.7	1.3	N	4	Yes	84	No
16	2.53	88.5	1.63	1.6	2.6	P	4	No	120	No
17	2.38	85.5	1.87	<0,1	1.9	M	4	No	132	No
18	2.84	84	1.77	0.7	1.2	P	4	No	72	No
19	2.48	48.3	1.87	<0.1	1.2	M	4	No	144	No
20	2.74	32.5	2.18	16.3	2.5	P	4	No	132	No
21	2.78	25.7	2.37	1.1	1.5	U	4	No	132	No
22	2.45	145.4	2.34	5.5	3	N	3	No	120	No
23	1.86	116.5	1.81	5.5	4.4	N	4	No	96	Yes
24	2.77	50.9	2.46	<1.0	6.1	N	4	No	60	Yes
25	2.76	17.4	2.44	7.7	1.7	P	4	Yes	72	No
26	1.93	129.5	2.47	3.3	5.9	N	4	No	60	Yes
27	2.64	126.3	2.30	<0.1	1.3	M	4	No	96	No
28	1.96	139.4	2.10	<0.1	2.7	M	4	Yes	72	No
29	2.57	33.5	2.36	1.4	1.3	P	3	Yes	144	No
30	2.84	23.8	2.27	5.1	1.9	P	2	Yes	72	No
31	2.64	74.7^a^	2.28	2	2.4	N	4	No	84	No
32	2.71	130	2.16	0.1	2.8	M	4	No	132	No
33	2.64	137	1.95	6.6	6	N	4	half	120	Yes
34	2.79	16	2.02	7	0.9	N	3	Yes	108	No
35	2.56	171	1.96	0.4	1.9	P	4	No	132	No
36	2.38	55.2^a^	1.91	1.2	1.2	P	4	Yes	84	No
37	2.66	18.3^a^	2.71	<0.3	2.4	M	4	No	48	No
38	2.72	164	2.15	15.6	9.1	P	4	No	120	Yes
39	2.82	52.1	2.45	5.5	1.2	U	4	No	72	No
40	2.81	69.1^a^	2.59	1.2	7.6	P	4	half	48	Yes
41	2.85	249.6	1.98	0.5	1.3	P	4	half	120	No
42	2.60	79.7	2.26	4.4	1.1	P	4	No	108	No

*PTX* parathyroidectomy, *PTH* intact parathyroid hormone, ^a^patients receiving cinacalcet before surgery, *P* positive = PTH decrease of more than 50 % of the pre-ischemic value, *N* negative = PTH decrease of less than 20 % of the pre-ischemic value, *U* source of PTH unpredictable = PTH decrease more than 20 % and less than 50 % of the pre-ischemic value, *M* Casanova-test meaningless, *rej*. rejection of the transplant, *comb* combined pancreas and kidney transplant

**Table 2:** Outcome of parathyroidectomy with autotransplantation into the tibialis muscle

	Number of patients			
	Preoperative	Intraoperative	Postoperative	Follow up
Hypopara (PTH ≤0.3 pmol/l)		0	17	7
Reduced PTH (PTH >0.3 - <1.3 pmol/l)		2	8	8
Eupara (PTH 1.3-7.6 pmol/l)		19	10	20
Elevated PTH (PTH >7.6 pmol/l)	42	21	2	4
Hyperpara according KDOQI [20]				3
Mean PTH (range 1.3-7.6 pmol/l)	84.0 ± 56.2	10.6 ± 9.1	1.38 ± 2.4	5.42 ± 10.9
Mean Calcium (range 2.1-2.65 pmol/l)	2.60 ± 0.2	Nodata	2.1 ± 0.3	2.17 ± 0.3

*PTH* intact parathyroid hormone

In conclusion, TPTX with AT into tibialis anterior muscle was successful in 86 % of the patients regarding PTH levels and 96 % regarding calcium levels.

### Modified Casanova-test

The modified Casanova-test was well tolerated by all patients with some reporting a feeling of numbness and mild pain during the ischemic time, and was discontinued in 3 patients after 10 min (7 %). No other side effects were reported.

**Fig. 1:**
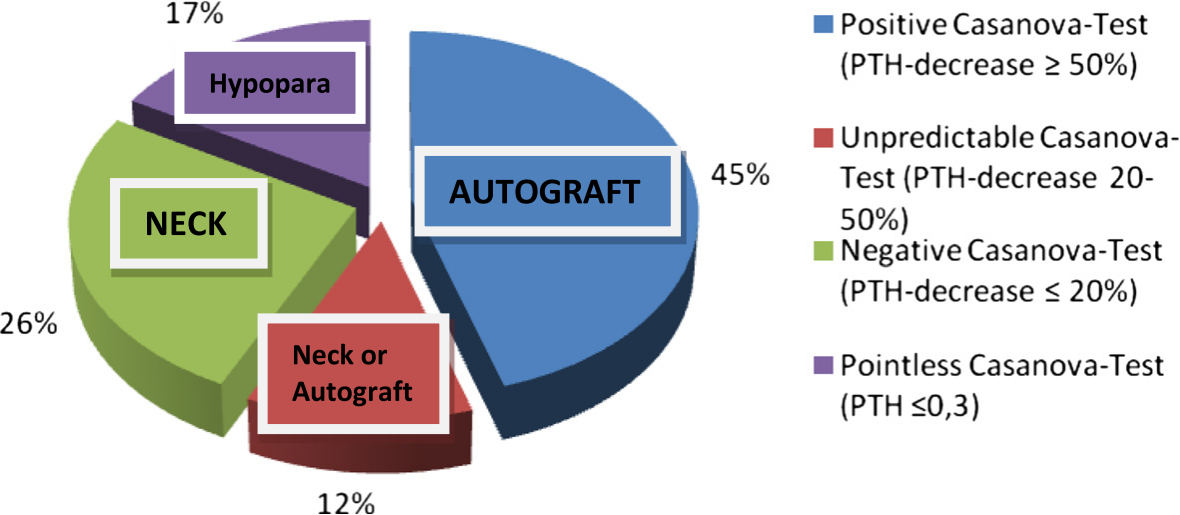
Casanova-Test and source of parathyroid hormone. Casanova-Test results show a positive Casanova-Test (PTH decrease of more than 50 % of the pre-ischemic value) in 19 patients (45 %, source of PTH: autograft), a negative Casanova-Test (PTH decrease of less than 20 % of the pre-ischemic value) in 11 patients (26 %, source of PTH: neck), an unpredictable Casanova-Test (PTH decrease more than 20 % and less than 50 % of the pre-ischemic value) in 5 patients (12 %, source of PTH unidentifiable: neck or autograft) and a pointless Casanova-Test (pre-ischemic value of PTH ≤ 0.3 pmol/l) in 7 patients (17 %, biochemical hypoparathyroidism). *PTH: intact parathyroid hormone; Hypopara: biochemical hypoparathyroidism*

The source of PTH secretion was assessed by comparing PTH values at 15 min of ischemia (or at 10 min when the former were not available) to the pre-ischemic values. 19 patients (45 %) had a significant drop in PTH (≥50 %); 11 patients (26 %) had a PTH drop of ≤20 % of pre-ischemic value. The source of PTH was classified as unidentifiable (PTH drop >20 % but <50 %) in five patients (12 %). No meaningful results were observed for seven patients with undetectable PTH levels (Fig. 1). The 50 %-drop in PTH that classified the parathyroid autograft as the source of the PTH secretion was obtainable with 95 % accuracy (in 18 of the 19 patients) after 10 min of temporary ischemic blockage.

Among the 19 patients that were positive for Casanova-test, 10 patients had normal PTH levels, as a sign for an intact graft function; four patients had higher than normal PTH levels (only two higher than the KDOQI recommended levels), indicating that the autograft was the dominant source of the elevated PTH; five patients had lower than normal PTH levels, suggesting a limited graft function. When blood flow was restored PTH levels increased up to basal values.

Among the 11 patients that were negative for Casanova-test, seven patients had normal PTH levels, as a sign for remaining parathyroid tissue or an accessory parathyroid gland in the neck/mediastinum; two patients had PTH levels higher than normal (only one patient higher than the recommended KDOQI levels) indicating the dominant source of the elevated PTH to be cervical/mediastinal. Two patients had PTH levels lower than normal, suggesting an insufficient PTH secretion of an in-situ parathyroid gland in the neck/ mediastinum. Among these 11 negative Casanova-tests, three patients had undergone a limited parathyroidectomy of less than four glands, three patients had undergone a partial and two a total thymectomy.

In the remaining five cases (unpredictable source of PTH secretion), three patients had normal PTH levels, one patient had PTH levels higher than normal but not higher than the recommended KDOQI levels and one patient had PTH levels lower than normal. The intermediate drop of PTH indicates functioning residual cervical/mediastinal glands additional to an active autograft. Among these five undefined Casanova-test results, no patient had undergone a limited parathyroidectomy of less than four glands, one patient had undergone a partial and one a total thymectomy.

In conclusion, 57 % of the patients had a functioning autograft according to the Casanova-test.

## Discussion

Parathyroid autotransplantation has been recorded in the modern scientific literature since the beginning of the 20^th^ century [21]. Definitive evidence that transplanted parathyroid glands function was gathered by Wells et al. [10], who introduced the parathyroid autotransplantation into the forearm [11]; this approach such as other AT sites have been favored ever since [7, 9].

In this study, based on our traditional understanding of the key features in successful parathyroid autografting in sHPT, the tibialis anterior muscle was shown to be a novel viable site of AT of the parathyroid tissue after TPTX. Autotransplantation into the tibialis anterior muscle was successful in 86 % of the patients with respect to PTH levels and 96 % regarding calcium levels. 17 % of the patients had postoperative biochemical hypoparathyroidism, however only 7 % were symptomatic and only 2 % suffered from severe hypocalcemia. This is comparable to previous studies which have reported the rate of hypoparathyroidism in 30 % after TPTX + AT, when gland was transplantated to other sites than tibialis anterior muscle [22].

As the remaining vital parathyroid tissue stays exposed to the same pathophysiological environment, graft-dependent recurrent disease is a common complication after TPTX with AT, resulting in further diagnostic tests and reoperative explorations. Recurrence has been reported in 4 to 30 % of patients in recent studies and 45 to 80 % of patients in older studies [2, 5, 23–26], although the risk of recurrence may be minimized by selecting the appropriate tissue [3, 22, 27–29]. Tominaga et al. reported a graft-dependent recurrence of 17.4 % in a study cohort of 2660 patients [30]. In the current study, only 5 % of the patients developed graft-dependent recurrent HPT according to the KDOQI recommended PTH levels [20], however without therapeutic consequences.

The assessment of graft function by PTH blood sampling is routinely reported in the literature [2, 4–6, 15], as is calcium levels [2, 31]. However both methods cannot be used to assess the presence of a supernumerary gland or ectopic parathyroid tissue. Only one study has attempted to examine the actual parathyroid autograft function using the Casanova-test [19]. The autograft function may be also indirectly assessed by measuring the level of PTH in sera from the antecubital veins of the grafted and nongrafted arms, with a PTH ratio of 1.5 or greater indicating functional parathyroid tissue [14, 32]. The percentage uptake of parathyroid tissue autografts using Wells’ method has been reported to range from 35 to 96 % [32–35]. The percentage uptake of subcutaneously or intramuscular injected parathyroid tissue is comparable (69–90 %), however in a relatively short follow-up period [13, 14, 16]. In the current study, 57 % of the patients had a functioning autograft in a long-term follow-up according to the Casanova-test; 26 % of the patients had a satisfactory PTH secretion without a positive Casanova-test, indicating active remaining parathyroid tissue within the neck/mediastinum.

Besides allowing easy surgical access and simple testing, the implantation of parathyroid tissue into the lower limb could be advantageous as compared to the forearm, since in dialysis patients the anatomy of the forearms is often changed due to arteriovenous fistula (AVF) operations [36, 37]. In addition, surgical revision of the fistulas or replacement with a graft, which is necessary in cases of AVF failure, stenosis, dysfunction or occlusion, could be more complicated after autotransplantation of parathyroid tissue. Early AVF failure is common, with an incidence in the range of 20 to 60 % [38, 39]. The tibialis anterior muscle is a relatively large and resistant muscle and therefore enables wide excisions without sacrificing vital structures, if infiltrating growth of parathyroid graft were to occur. Since dialysis patients often have a difficult vascular situation, it appears important to protect the forearm for an additional shunt revision or replacement.

Since this study was intended as pilot study, no control group was included. A further limitation of the study is that the surgical procedure wasn’t standardized. Routine transcervical thymectomy was not performed. Finally, the function of the graft might be influenced by various factors, e.g. 33 % of patients with insufficient PTH secretion had diabetes mellitus of which 80 % revealed undetectable PTH levels (*p* <0.05). Further studies with larger number of patient cohorts are required to confirm these data.

## Conclusions

Total parathyroidectomy with AT of parathyroid tissue in the lower limb is a safe and effective therapy for renal HPT. The autograft function could be effectively assessed by means of the modified Casanova-test The advantage of preserving the forearm in CRI patients combined with the easy testing of autografts in extremities qualifies this procedure as an alternative therapy for sHPT.

## Abbreviations

AT: Autotransplantation
AVF: Arteriovenous fistula
CRI: Chronic renal insufficiency
ESRD: End stage renal disease
HPT: Hyperparathyroidism
KDOQI: Kidney Disease Outcomes Quality Initiative
PTH: Intact parathyroid hormone
SCAT: Simplified Casanova autografectomy test
sHPT: secondary hyperparathyroidism
TPTX: Total parathyroidectomy
TPTX + AT: Total parathyroidectomy with autotransplantation
